# Association of Nogo-A Gene Polymorphisms with Cerebral Palsy in Southern China: A Case-Control Study

**DOI:** 10.1159/000527801

**Published:** 2022-11-02

**Authors:** Yuxin Wang, Lu He, Jingyu Huang, Jinling Li, Liru Liu, Yunxian Xu, Tingting Peng, Xubo Yang, Yiting Zhao, Chaoqiong Fu, Shiya Huang, Hongmei Tang, Kaishou Xu

**Affiliations:** Department of Rehabilitation, Guangzhou Women and Children's Medical Center, Guangzhou Medical University, Guangzhou, China

**Keywords:** Cerebral palsy, Nogo-A, Single-nucleotide polymorphisms, Genotyping, Central nervous system

## Abstract

Cerebral palsy (CP) is a motor and postural disorder syndrome caused by the nonprogressive dysfunction of the developing brain. Previous studies strongly indicated that the Nogo-A gene might be related to the pathogenesis of CP. The objective of this research was to explore the relationship between Nogo-A polymorphisms (rs1012603, rs12464595, and rs2864052) and CP in Southern China. The Hardy-Weinberg equilibrium (HWE) testing, allele and genotype frequencies analysis, and haplotype association analysis were applied to the genotyping of 592 CP children and 600 controls. The results showed that the allele and genotype frequencies of rs1012603 of CP group were significantly different from the control group. The haplotype “TTGGG” was significantly associated with an increased risk of CP. The allele frequencies of rs1012603 were significant differences between CP with spastic diplegia, female CP cases, and controls. Furthermore, significant differences in allele and genotype frequencies were also noticed between GMFCS I of CP and controls for rs1012603, and significant differences in allele and genotype frequencies were observed between the ADL (>9) of CP and controls for rs1012603 and rs12464595. This study showed that the SNPs rs1012603 of Nogo-A were significantly correlated with CP, and the correlations were also found in spastic diplegia, GMFCS I of CP, ADL (>9) of CP, and female subgroups, indicating that Nogo-A might mainly affect mild types of CP and there might be sex-related differences.

## Introduction

Cerebral palsy (CP) is a syndrome that involves motor and postural disorders arising from the nonprogressive dysfunction of the developing brain. Children with CP are frequently affected by epilepsy, secondary musculoskeletal problems, and related impairments, which may include disorders of sensation, cognition, or other brain functions [1, 2, 3]. CP is regarded as a permanent disability that influences the life expectancy and quality of life of patients, places economic pressure on parents, and has large social support costs [4, 5]. The natality of CP is 2–2.5 instances every 1,000 live births, despite extraordinary progress in clinical treatment and newborn care, the overall prevalence in different groups has not changed greatly in the last few decades [6].

The etiology of CP has been shown to be complex, including risk factors of premature birth, fetal growth retardation, infections, and other types of exposures during pregnancy [1, 5, 6]. However, a proportion of CP cases do not have definitive risk factors related to prepregnancy, pregnancy, and the perinatal period. Recent studies have suggested that genes also have important contributions to CP [7, 8]. A new exon sequencing study of the CP cohort found that, similar to other neurodevelopmental disorders, children with CP had a 32.7% incidence of pathogenicity and possible variants in hundreds of genes [9]. More and more evidences showed that CP is similar to mental disability, autism spectrum disorders, epilepsy, and other neurodevelopmental disorders [10, 11, 12]; some cases might be secondary to rare effect gene mutations, including copy number variations (CNVs) and single-nucleotide polymorphisms (SNPs) [13]. In the previous cohort study of CP, the positive rate of CNVs detected by chromosome microarray analysis was 9.6–31.0% [14, 15], and the positive rate of SNPs determined by exome sequencing was 14–19% [16]. Several kinds of potential disease-causing gene variants have been discovered, including those involved in inflammation and coagulation factors, and their potential association with CP has been demonstrated [17, 18, 19, 20]. Whole-exome sequencing revealed several potential genetic variations that contributed to CP. For example, the apolipoprotein genotype and inducible nitric oxide synthase-231 have been extensively studied. However, these findings require further functional and pathway researches for validation [7, 21, 22, 23], genetic variants are heterogeneous, and functional studies are required to confirm causality [24, 25].

The SNPs are the densest genetic markers in the human genome with a high frequency of occurrence, and they are considered to be the next generation of genetic markers after microsatellites. *Autophagy-related gene 7* (*ATG7*) [26], anti-inflammatory chemokine CCL18 [27], inducible NO synthase NOS2A [28], sex-related *interleukin-6* (*IL-6*) gene [29], nitric oxide synthase 1 (NOS1) [30], and other genes have been widely confirmed to be significantly associated with the occurrence and development of CP. Considering that the positive rate of SNPs in CP is 14–19% [16], there are more potential SNPs variants that need to be further discovered and studied. Our team's previous studies found that neurite outgrowth inhibitor-A (Nogo-A) plays a vital role in the nerve remodeling and functional recovery of CP mice [31], but so far, there is no research to explore the role of Nogo-A gene in the development of CP.

Nogo-A is a transmembrane protein of 1,200 amino acids (aa) that contains a C-terminal 200-aa reticulon domain. Nogo-A is associated with the inhibitory effect of axon regeneration after central nervous system (CNS) injury, which induces cytoskeleton collapse and inhibits neuronal growth mechanisms in combination with various receptors of cellular surface and activated complex signals transduction cascades [32, 33]. Nogo-A has also been proven to modulate neurogenesis, migratory, and contact-dependent processes during nervous system development [34]. An improvement in axonal growth and the enhancement of neural function after injury were connected with neutralizing or recovering Nogo-A receptor plasticity after injury, and this modulation did not have significant side effects [35]. There was a considerable increase in the level of Nogo-A in oligodendrocytes in lesions 2 months after cerebral ischemia in monkeys [36]. Another finding from a trial on the mice hippocampus indicated that Nogo-A restricted the induction and reactivation of long-term potentiation in the short term and the long term, which affected the recovery of both function and structure [35, 37].

The occurrence of CP in children is affected by many factors. The development of vascular and nerve function is closely related to the prognosis of CP [30]. Nogo-A, as a stabilizer and modifier of neural networks, acts as a crucial factor in the migration and growth of developing nerves [33]. A study in mice in which the Nogo-A gene was switched off reported that the number of capillaries and capillary branches in the brain increased, which might affect angiogenesis-dependent diseases in the CNS, including brain tumors or strokes [38]. To some extent, Nogo-A has been shown to be essential for vascular development and cerebral circulation [38]. In addition, previous research has revealed that Nogo-A is involved in some neurologic disorders such as apoplexy, Alzheimer's disease, multiple sclerosis, and schizophrenia [39]. These results strongly indicate that the Nogo-A gene may be related to the pathogenesis of CP, though the specific biological role of Nogo-A in the etiopathogenesis of CP is still unclear. Therefore, the objective of this research was to explore the relationship between Nogo-A gene polymorphisms and CP in Southern China to provide new ideas for the study of the mechanism of CP gene level and to provide theoretical support for the personalized treatment of CP.

## Materials and Methods

### Research Population

**Fig. 1:**
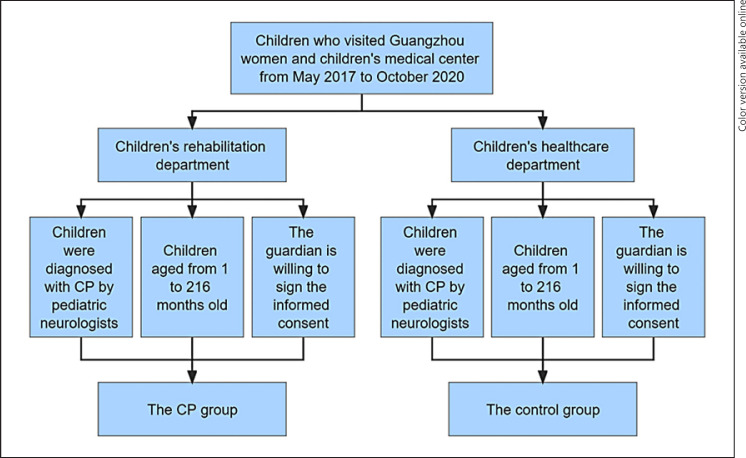
Inclusion criteria flowchart for study subject.

This was a case-control study of children recruited from Guangzhou Women and Children's Medical Center from May 2017 to October 2019, Southern China. The subjects in the control and CP groups were from the Departments of children's healthcare and rehabilitation, respectively. Participants were eligible for inclusion in the CP group if they were (1) diagnosed with CP by pediatric neurologists, according to clinical examinations and neuroimaging results and the guidelines published by the Surveillance of Cerebral Palsy in Europe (SCPE) [6], (2) aged from 1 to 216 months, (3) those whose guardians agreed to sign informed consent. The inclusion criteria of control group include (1) children with normal development who were diagnosed by pediatricians as free of CNS diseases, (2) aged from 1 to 216 months, (3) those whose guardian is willing to sign the informed consent. The flowchart of the inclusion criteria of the research subjects is shown in Figure 1. After screening, this study cohort included 592 CP patients and 600 children with normal development. There were 204 female (34.5%) and 388 male (65.5%) patients in the CP group, who ranged from 8 to 198 months old. The control group included 207 females (34.5%) and 393 males (65.5%), who were ranged from 1 to 206 months old. This study received authorization from the Ethics Review Board of Guangzhou Women and Children's Medical Center (No. 2016021619). And the investigation was conducted in accordance with the ethical standards of the World Medical Association Declaration of Helsinki.

The clinical data records for this study contained basic clinical information on the two groups, high-level perinatal factors, and evaluation results from children with CP. Basic clinical materials included sex, gestational age, and birth weight. The risk factors included premature delivery, low birth weight, neonatal asphyxia, hypoxic-ischemic encephalopathy (HIE), intracranial hemorrhage (ICH), neonatal jaundice, and maternal factors (including amniotic fluid abnormalities, multiple pregnancies, older age during pregnancy, and dystocia) (shown in Table 1). Based on the definition of the World Health Organization, premature birth refers to a gestational age of less than 37 weeks [39]. The standard for infant weight was formulated by the National Health Planning Commission, which states that normal birth weight infants have birth weights of more than or equivalent to 2,500 g and less than 4,000 g and that low birth weight infants are born weighing less than 2,500 g. The neonatal asphyxia was defined as hypoxemia, hypercapnia, metabolic acidosis, and multiple organ damage resulting from inability to establish normal spontaneous breathing after birth, the perinatal mortality was one of the leading causes of disability in children and adolescents, and its diagnostic criteria were based on the diagnostic criteria formulated by the American Academy of Pediatrics and the American College of Obstetricians and Gynecologists: (1) umbilical artery blood showed severe metabolic or mixed acidosis, pH value less than 7.0; (2) Apgar score 0–3 and duration greater than 5 min; (3) the newborn has nervous system manifestations in early stage, such as convulsion, coma, or hypotonia; (4) there was evidence of multiple organ dysfunction early in life. HIE was diagnosed by satisfying the following metabolic acidosis parameters in the initial several hours after birth: pH value of the umbilical cord blood less than 7.0; base deficit greater than 12 mEq/L; substantiation of respiratory support required in the first minute after birth; and low Apgar scores continuing after 5 min of birth [40]. The neonatal ICH was mainly diagnosed according to a cranial imaging examination and included periventricular-intraventricular hemorrhage, primary subarachnoid hemorrhage, parenchymal hemorrhage, subdural hemorrhage, and cerebellar hemorrhage [41]. The clinical subtypes of CP (spastic hemiplegia, spastic diplegia, spastic quadriplegia, dyskinetic, and mixed) were also recorded in the database. The Gross Motor Function Classification System (GMFCS) is regarded as a standard assessment tool to classify the motor function of CP patients, which reflected their motor ability [42]. The activity of daily life (ADL)-related tool S-M activities of daily living ability scale was used to evaluate the daily living standard of the CP patients, and the S-M activities of daily living ability scale (with 9 points being the cutoff level and higher scores indicating a higher living ability) was used for patients from infancy to junior-school age [43].

### SNP Selection and Genotyping

We used the Single Nucleotide Polymorphism Database (dbSNP) (https://www.ncbi.nlm.nih.gov/SNP) and the international HapMap project database (https://www.ncbi.nlm.nih.gov/variation/tools/1000genomes/) [29] to screen candidate SNPs. SNPs were limited to noncoding regions including 2,000 bp of Nogo-A upstream and downstream (3′-untranslated regions and introns); minor allele frequencies were not less than 20%; the selected SNPs showed low linkage disequilibrium (LD) (*R*^2^ < 0.8). All of the three SNPs are located in the intron of Nogo-A: rs1012603, rs12464595, and rs2864052.

We collected DNA samples from the blood of the two groups of children using a AxyPrep DNA Miniprep Kit (Axygen Biosciences, USA). Primer 3 (http://primer3.sourceforge.net/webif.php) was used to design probes and primers and to filter the results [29]. Polymorphic cross-fragments were amplified by multiplex polymerase chain reaction prior to using a MassARRAY system (http://www.sequenom.com) for genotyping. SpectroTyper software (Sequenom, Inc.) was used for genotype calling. A double-blind method was used for gene analysis and clinical data collection. In order to ensure the reliability and reproducibility of the experiment, we prepared a blank control in each 384-well plate, and randomly selected 10% of all samples for repeat genotyping, with the repeatability of 100%.

### Statistical Analysis

Statistical analysis was conducted using SPSS software (version 20.0, USA). The χ^2^ test or Fisher's exact test was used to analyze the allele and genotype frequencies of different SNPs between CP children and controls. The logistic regression analysis was performed to estimate the association between Nogo-A gene and risk of CP. Genetic analysis (including Hardy-Weinberg equilibrium [HWE] testing, allele and genotype frequencies analysis, LD analysis, and haplotype association analysis) was conducted by using SHEsis software (http://analysis.bio-x.cn/myAnalysis.php). Except for its role as representing the quality of the research, HWE was also used as a primary assumption for the reliability of per-allele analysis [44]. LD was assessed by the standardized disequilibrium coefficients D′ and *R*^2^, which indicated the information about the recombination and mutation history between allele frequencies in two groups [45]. The haplotype analysis fully considered the LD information for several loci and has been widely proven to be a convenient and effective method for candidate gene analysis. The associated risk factors were evaluated by the odds ratio (OR), and the 95% confidence interval (CI) was measured. A *p* value <0.05 was regarded as having statistical significance.

## Results

### Overall Analysis

**Fig. 2:**
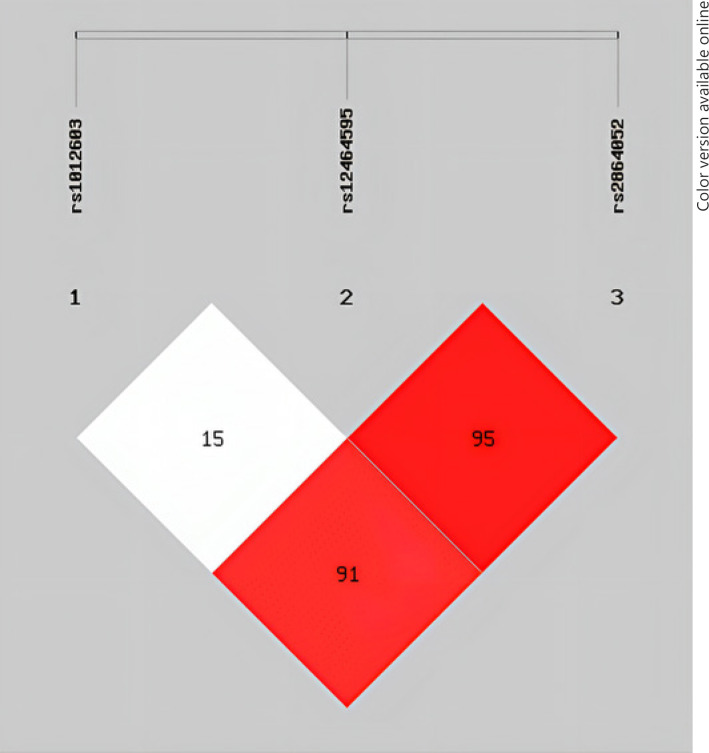
Linkage disequilibrium analysis of three SNPs in Nogo-A gene.

Before the genotype analysis, we tested whether the three SNPs deviated from HWE. The results showed that the genotype distribution of the three SNPs (rs1012603, rs12464595, and rs2864052) did not deviate from HWE when comparing the CP group to the control group (*p* > 0.05). Therefore, the three SNPs were retained for further testing (shown in Table 2). According to the allele and genotype frequencies analysis, the allele frequencies of rs1012603 were significant differences between the CP and control groups (rs1012603: OR = 0.783, 95% CI = 0.631∼0.971, *p* = 0.026) (shown in Table 2). And the genotype frequencies of rs1012603 were also significant associated with CP (*p* = 0.043) (shown in Table 2). For rs1012603, the CP group had a significantly higher allele T and CT genotype frequency compared to the control group. These results indicated that patients carrying the rs1012603 CT genotype had an increased risk of CP compared with those with other genotypes. The SNP pairs rs1012603/rs2864052, rs12464595/rs2864052 showed strong LD (D′ > 0.9) according to the LD analysis of the three SNPs, indicating potential interactions between them, which is shown in Figure 2.

In order to determine whether these three SNPs have good predictive value when analyzed together, we analyzed the haplotypes of these three SNPs. The haplotype analysis showed that there were significant differences between the CP and control groups for the three SNP haplotypes (*p* = 0.019), and the distribution of haplotype “TTGGG” in CP was significantly more than that in the control group (OR = 1.457, 95% CI = 1.153∼1.841, *p* = 0.002) (shown in Table 3), suggesting that this haplotype significantly increased the risk of CP.

### Subgroup Analysis

The occurrence of CP is affected by many factors. To verify the potential relationship between gender or high-risk factors (premature delivery, low birth weight, neonatal asphyxia, HIE, ICH, neonatal jaundice, and maternal factors) and genotypes; subgroup analyses of these factors were carried out. The results of subgroup analysis demonstrated significant differences in the allele frequencies of rs1012603 between the female CP cases and the control group (*p* = 0.043). Nevertheless, there was no significant difference between the other hazard factors for CP and the control group with regard to the allele and genotype frequencies analysis.

It is worth noting that the clinical classification and functional grading of CP are directly related to the prognosis of patients. We compared the genotypes of different subtypes of CP and different functional grading of CP (GMFCS and ADL) with the control group, which helps provide guidance for clinical interventions for CP. The allele frequencies of rs1012603 were significant differences between CP with spastic diplegia and controls (OR = 0.749, 95% CI = 0.579–0.969, *p* = 0.028) (Table 4). Furthermore, we found that there were significant differences in the allele frequencies of rs1012603 between the GMFCS I of CP and controls (OR = 0.711, 95% CI = 0.536–0.942, *p* = 0.017) (Table 5). And the genotype frequencies of rs1012603 were also significant differences between the GMFCS I of CP and controls (*p* = 0.027) (shown in Table 5). Furthermore, significant differences in allele and genotype frequencies were observed between the ADL (>9) of CP and controls for rs1012603 (p_allele_ = 0.007, p_genotype_ = 0.013) and rs12464595 (p_allele_ = 0.012, p_genotype_ = 0.043) (shown in Table 6). No significant correlations in genotypes were found among the control group and the other clinical typing or functional grading of CP in the subgroup analyses.

## Discussion

This study was the first case-control research to analyze genetic variation of the Nogo-A gene in 600 controls and 592 CP children. Our results showed that the rs1012603 of Nogo-A was significantly correlated with CP, and the correlations were also found in spastic diplegia, GMFCS I of CP, ADL (>9) of CP, and female subgroups, indicating that Nogo-A might mainly affect mild types of CP and there might be sex-related differences. These findings provided new ideas and perspectives for the study of the mechanism of CP gene level.

The pathogenesis of CP is extremely complex, although many high-risk factors such as premature birth, infection, hypoxia ischemia, prenatal, and perinatal stroke have been confirmed to be the important factors leading to CP [1]. But so far, no single pathogenic factor of CP has been found. As many as 40% of CP cases may not have an easily identifiable cause [9], therefore, they are defined as cryptogenic or idiopathic CP [10]. The latest epigenetic studies have shown that mutations in a series of gene loci were closely related to the occurrence and development of CP, such as NM_016824.5 in the ADD3 [46], the mutation of *ARG1* [47] and *GPAM* genes [48]. Multiple studies have shown that CNVs and SNPs were significantly associated with CP [11, 12, 14, 15]. Recently, researchers have conducted extended studies on these early findings and found that destructive SNPs were independent risk factors for CP, and this conclusion was supported by reliable statistical evidence at the cohort level [16].

The aa sequence of Nogo-A was identified as early as 2,000 [30]. Human Nogo-A gene belonged to reticulin encoding gene family, which was about 75 kb long and located on chromosome 2P16.1 [49]. Nogo-A is a high-molecular weight membrane protein [30, 49] widely distributed on the surface of oligodendrocytes and neurons, the primary role of which is to control the rearrangement of the cytoskeleton, protein synthesis, and gene expression in neurocytes by activating specific receptors [50]. In addition, Nogo-A has an inhibitory effect on neurite growth in the early stage of development [51], which affecting the migration of nerve cells in early neural tube [52, 53]. Nogo-A affected amyloid deposition and the formation of dystrophic neurites by negatively regulating BACE1 activity, thereby affecting the metabolism of Aβ and triggering the occurrence and development of Alzheimer's disease [54]. Xu et al. confirmed that Nogo-A might be involved in the development of tuberous sclerosis syndrome and focal type 2b cortical disease or seizures by mediating the NgR/LINGO-1/TROY signal transduction pathway [55].

Our results showed that the allele and genotype frequencies of rs1012603 were significantly correlated with CP. The most common pathological brain changes in CP have been demonstrated to be periventricular leukomalacia, cerebral circulation dysfunction, and underdeveloped mechanism of cerebrovascular self-regulation [56, 57]. Moreover, neuropathological features of periventricular leukomalacia included activation of microglia and focal and diffuse abnormalities of oligodendrocytes [58]. The perinatal brain is at a critical period of increased axon synapse formation and myelination [59]; any imbalance of growth factor or inhibitory factor will have an important impact on perinatal brain. Previous studies and our team's previous studies have suggested that neurological diseases (such as stroke, CP) could significantly increase the expression of Nogo-A and its effector protein in the brain [37, 60]. Eslamboli et al. [34] demonstrated a significant increase in the number of Nogo-A immunopositive oligodendrocytes in the white matter region of cerebral ischemic marmosets. The abnormal level of Nogo-A has a negative effect on the brain development of perinatal fetus through a series of mechanisms. It might destabilize actin filaments and inhibit the extension of axons and the movement of growth cones by activating RhoA (the central mediator of cytoskeleton rearrangement). In addition, Nogo-A might inhibit the expression of neuronal growth genes [61] by downregulating cAMP response element-binding protein phosphorylation [62]. The migration of developing nerve cells was blocked, which delayed the formation of axon synapses and myelin sheaths, indirectly affected the development of white matter in the brain. On the other hand, Nogo-A served a negative regulatory function in angiogenesis by restricting the migration of vascular endothelial cells, which seriously affected cerebrovascular development and circulation. Previous studies have shown that its deletion could significantly promote vascular germination and the repair of the ischemic CNS [63, 64]. Therefore, the SNPs rs1012603 of Nogo-A might engage in the regulation of Nogo-A expression by regulating the stability and translation of mRNA and regulating the interaction between proteins, thereby indirectly affect the formation of axon synapse and myelin sheath in the white matter, and hinder the development and circulation of cerebral blood vessels, which jointly promoted the onset and development of CP.

An increasing number of studies have revealed that different genetic factors may be correlated with diverse subtypes of CP [65, 66]. For instance, most subtypes of CP that involve mental retardation are induced by recessive variations in the genes of 4 protein-binding complexes (AP4M1, AP4E1, AP4S1, and AP4B1) [67, 68, 69]. Previous studies have shown that rs1470612 and rs2594972 SNPs in autophagy-related gene 7 are associated with CP, and stronger correlation is found in spastic paraplegia and male subgroups [26]. Yu T found that *nitric oxide synthase 1* (*NOS1*) gene was associated with spastic quadriplegia and CP with neonatal encephalopathy [29]. But so far, there was no study to explore the role of Nogo-A in CP. Based on our previous research on Nogo-A [37], this study further discovered that the rs1012603 SNPs in Nogo-A were significant differences between CP with spastic diplegia, GMFCS I of CP, the ADL (>9) of CP, the female subgroups, and controls, which implied that the Nogo-A mainly affected mild CP children and there might be sex-related differences. The reason for this result might be that spastic diplegia was the most common subtype of CP, accounted for 54.9% of CP [56, 70, 71]. Although so far, the gene expression and function changes caused by our selected loci have not been reported. The rs1012603 of Nogo-A may have genetic variations (aa sequence changes [72], truncation mutations, stop codons [73], frameshift mutations [74]) in children with CP, which may lead to changes in the expression of Nogo-A and play a negative role in regulation expression and function of the Nogo-A. In the follow-up study, we will further verify which mutations exist at the rs1012603 through genetic testing and investigate which form causes the expression changes of the Nogo-A. And there was obvious heterogeneity among the study populations. So, the result needed to be verified in a larger sample size.

Although our research results show that there is no statistically significant correlation between high-risk factors and CP, Nogo-A might not necessarily be a main cause of CP progression in our research but have interacted with potential factors (such as perinatal high-risk factors and clinical and environmental variables) to collectively cause CP. Deng et al. [49] confirmed that the release of glutamate, free radicals, and pro-inflammatory cytokines were also important triggers of CP. The more severe the type of CP is, the more complicated the pathogenesis involved may be.

Notably, some limitations existed in our study that needed to be improved in further researches. For example, only three SNPs of the Nogo-A gene were explored in this research, and more Nogo-A variants should be screened to explore the connection between the Nogo-A gene and CP in a more comprehensive manner in future work. In addition, taking familial genetic factors into account may be more conducive to the functional study of the Nogo-A gene in the occurrence and development of CP. More rigorous and specific verification should be explored in animal models to ascertain the role of the expression of Nogo-A in the diverse stages of CP progression to provide more powerful evidence to aid in the rehabilitation of CP. A complete assessment of the Nogo-A gene in CP requires a larger sample size, and the results of these experiments need to be verified in a larger population sample to provide guidance for clinical interventions. Proteomics complements the shortcomings of genomics and transcriptomics to explore the structure and function of specific proteins from the perspective of clarifying the protein identity of organisms [75]. In the follow-up study, we will continue to further explore the difference in the expression of Nogo-A protein between CP and control children, in order to provide new ideas and perspectives for the etiological study of CP. In summary, this study showed that the SNPs rs1012603 of Nogo-A were significantly correlated with CP, and the correlations were also found in spastic diplegia, GMFCS I of CP, ADL (>9) of CP, and female subgroups, indicating that Nogo-A might mainly affect mild types of CP and there might be sex-related differences, which provides novel evidence for the role of Nogo-A in CP and contributes to our understanding of the molecular mechanisms of this neurodevelopmental disorder.

## Statement of Ethics

The investigation was conducted in accordance with the ethical standards of the World Medical Association Declaration of Helsinki. This study received authorization from the Ethics Review Board of Guangzhou Women and Children's Medical Center (No. 2016021619). And all participants' parents or legal guardians signed informed consent forms.

## Conflict of Interest Statement

The authors have no conflicts of interest to declare.

## Funding Sources

The author(s) disclosed receipt of the following financial support for the research, authorship, and/or publication of this article: this work was supported by the National Natural Science Foundation of China [Grant No. 81902309, 81672253]; the Natural Science Foundation of Guangdong Province [Grant No. 2021A1515012543, 2019A1515010420]; the Science Technology and Innovation Development Foundation of Guangzhou [Grant No. GWCMC2020LH-1-004]; and Featured Clinical Technique Foundation of Guangzhou [Grant No. 2019TS55]. The funders had no role in the study design, data collection and analysis, decision to publish, or preparation of the manuscript.

## Author Contributions

Kaishou Xu conceived this project. Hongmei Tang coordinated this project. Yuxin Wang wrote the manuscript. Lu He identified candidate sites and Jinling Li designed the experiments. Jingyu Huang recruited the control group cohort and Liru Liu recruited the CP group cohort. Yiting Zhao collected medical information. Chaoqiong Fu and Shiya Huang participated in the collection and processing of cohort samples, respectively. Tingting Peng assessed the functional grading of patients. Xubo Yang performed the genetic analysis. Yunxian Xu conducted the statistical and bioinformatics analysis. All authors read and approved the final manuscript.

## Data Availability Statement

All data generated or analyzed during this study are included in this article. Further inquiries can be directed to the corresponding author.

## Figures and Tables

**Table 1:** Clinical characteristics of all participants

Characteristic	Controls (*n* = 600)	CP patients (*n* = 592)
Sex		
Male/female	393/207	388/204
Gestational age		
≥37 weeks	−	284
<37 weeks	−	308
Birth weight		
≥2,500 g	−	344
<2,500 g	−	248
Neonatal asphyxia		
Yes	−	79
No	−	513
HIE		
Yes		60
No		532
ICH		
Yes	−	39
No	−	553
Neonatal jaundice		
Yes	−	42
No	−	550
Maternal		
Yes	−	35
No	−	557
Subtype of CP		
Spastic hemiplegia	−	183
Spastic diplegia	−	300
Spastic quadriplegia	−	83
Dyskinetic	−	14
Mixed	−	12
GMFCS level		
I	−	220
II	−	202
III	−	111
IV	−	46
V	−	13
ADL		
ADL >9	−	247
ADL ≤9	−	345

CP, cerebral palsy; HIE, hypoxic-ischemic encephalopathy; ICH, intracranial hemorrhage; GMFCS, the Gross Motor Function Classification System; ADL, activity of daily life (with 9 points being the cutoff level and higher scores indicating a higher living ability).

**Table 2:** Allele and genotype frequencies of SNPs in CP and controls

Group	Allele frequency		*p* value	OR (95% CI)	Genotype frequency			*p* value	HWE
	C	T			C/C	C/T	T/T		P
rs1012603									
Control	1,019 (0.849)	181 (0.151)	**0.026**	0.783	427 (0.712)	165 (0.275)	8 (0.013)	**0.043**	0.072
CP	965 (0.815)	219 (0.185)		(0.631–0.971)	391 (0.660)	183 (0.309)	18 (0.030)		0.539
rs12464595									
Control	193 (0.161)	1,007 (0.839)	0.218	0.869	22 (0.037)	149 (0.248)	429 (0.715)	0.107	0.050
CP	169 (0.143)	1,015 (0.857)		(0.694–1.087)	10 (0.017)	149 (0.252)	433 (0.731)		0.489

	A	G			A/A	A/G	G/G		P
rs2864052									
Control	447 (0.372)	753 (0.627)	0.607	0.957	83 (0.138)	281 (0.468)	236 (0.393)	0.874	0.965
CP	429 (0.362)	755 (0.638)		(0.810–1.131)	78 (0.132)	273 (0.461)	241 (0.407)		0.960

CP, cerebral palsy; SNPs, single-nucleotide polymorphisms; CI, confidence interval; HWE, Hardy-Weinberg equilibrium test.

**Table 3:** Haplotype analysis between CP cases and controls

Haplotype	Case (frequency)	Control (frequency)	*p* value	OR (95% CI)
C C G A G*	126.30 (0.107)	153.63 (0.128)	0.111	0.815 (0.634–1.049)
C T A A A*	392.58 (0.332)	426.74 (0.356)	0.233	0.900 (0.757–1.070)
C T G A A*	297.94 (0.252)	309.93 (0.258)	0.749	0.970 (0.805–1.169)
C T G A G*	81.51 (0.069)	77.82 (0.065)	0.677	1.071 (0.776–1.479)
T T G G G*	194.62 (0.164)	143.97 (0.120)	**0.002**	**1.457 (1.153–1.841)**
Global result			**0.019**	

CP, cerebral palsy; CI, confidence interval. Loci chosen for haplotype analysis: rs1012603, rs12464595, rs2864052 (frequency <0.03 in both controls and cases was dropped).

**Table 4:** Allele and genotype frequencies of SNPs in spastic diplegia and controls

Group	Allele frequency		*p* value	OR (95% CI)	Genotype frequency			*p* value
	C	T			C/C	C/T	T/T	
rs1012603								
Control	1,019 (0.849)	181 (0.151)	**0.028**	0.749	427 (0.712)	165 (0.275)	8 (0.013)	0.067
CP	485 (0.808)	115 (0.192)		(0.579–0.969)	193 (0.643)	99 (0.330)	8 (0.027)	
rs12464595								
Control	193 (0.161)	1,007 (0.839)	0.552	0.921	22 (0.037)	149 (0.248)	429 (0.715)	0.726
CP	90 (0.150)	510 (0.850)		(0.702–1.208)	8 (0.027)	74 (0.247)	218 (0.727)	

	A	G			A/A	A/G	G/G	
rs2864052								
Control	447 (0.372)	753 (0.627)	0.863	1.018	83 (0.138)	281 (0.468)	236 (0.393)	0.938
CP	226 (0.377)	374 (0.623)		(0.831–1.246)	44 (0.147)	138 (0.460)	118 (0.393)	

CP, cerebral palsy; SNPs, single-nucleotide polymorphisms; CI, confidence interval.

**Table 5:** Allele and genotype frequencies of SNPs in GMFCS I of CP and controls

Group	Allele frequency		*p* value	OR (95% CI)	Genotype frequency			*p* value
	C	T			C/C	C/T	T/T	
rs1012603								
Control	1,019 (0.849)	181 (0.151)	**0.017**	0.711	427 (0.712)	165 (0.275)	8 (0.013)	**0.027**
CP	352 (0.800)	88 (0.200)		(0.536–0.942)	140 (0.636)	72 (0.327)	8 (0.036)	
rs12464595								
Control	193 (0.161)	1,007 (0.839)	0.148	0.792	22 (0.037)	149 (0.248)	429 (0.715)	0.305
CP	58 (0.132)	382 (0.868)		(0.577–1.087)	4 (0.018)	50 (0.227)	166 (0.755)	

	A	G			A/A	A/G	G/G	
rs2864052								
Control	447 (0.372)	753 (0.627)	0.239	0.871	83 (0.138)	281 (0.468)	236 (0.393)	0.467
CP	150 (0.341)	290 (0.659)		(0.693–1.096)	24 (0.109)	102 (0.464)	94 (0.427)	

CP, cerebral palsy; SNPs, single-nucleotide polymorphisms; CI, confidence interval; GMFCS, the Gross Motor Function Classification System.

**Table 6:** Allele and genotype frequencies of SNPs in the ADL (>9) of CP and controls

Group	Allele frequency		*p* value	OR (95% CI)	Genotype frequency			*p* value
	C	T			C/C	C/T	T/T	
rs1012603								
Control	1,019 (0.849)	181 (0.151)	**0.007**	0.691	427 (0.712)	165 (0.275)	8 (0.013)	**0.013**
CP	393 (0.796)	101 (0.204)		(0.528–0.905)	155 (0.628)	83 (0.336)	9 (0.036)	
rs12464595								
Control	193 (0.161)	1,007 (0.839)	**0.012**	0.667	22 (0.037)	149 (0.248)	429 (0.715)	**0.043**
CP	56 (0.113)	438 (0.887)		(0.485–0.917)	3 (0.012)	50 (0.202)	194 (0.785)	

	A	G			A/A	A/G	G/G	
rs2864052								
Control	447 (0.372)	753 (0.627)	0.697	1.044	83 (0.138)	281 (0.468)	236 (0.393)	0.479
CP	189 (0.383)	305 (0.617)		(0.841–1.295)	31 (0.126)	127 (0.514)	89 (0.360)	

CP, cerebral palsy; SNPs, single-nucleotide polymorphisms; CI, confidence interval; ADL, activity of daily life.
